# The roles of lithium-philic giant nitrogen-doped graphene in protecting micron-sized silicon anode from fading

**DOI:** 10.1038/srep15665

**Published:** 2015-10-26

**Authors:** Xiaoxu Liu, Dongliang Chao, Qiang Zhang, Hai Liu, Hailong Hu, Jiupeng Zhao, Yao Li, Yizhong Huang, Jianyi Lin, Ze Xiang Shen

**Affiliations:** 1 Key Laboratory for Photonic and Electric Bandgap Materials, Ministry of Education, Heilongjiang University of Science and Technology, Harbin, Heilongjiang, China; 2 School of Physical and Mathematical Sciences, Nanyang Technological University, 50 Nanyang Avenue, 637371, Singapore; 3 School of Materials Science and Engineering, Nanyang Technological University, 50 Nanyang Avenue, 639798, Singapore; 4 Harbin Institute of Technology, Harbin, 150080, Heilongjiang, China

## Abstract

A stable Si-based anode with a high initial coulombic efficiency (ICE) for lithium-ion batteries (LIB) is critical for energy storage. In the present paper, a new scalable method is adopted in combination with giant nitrogen-doped graphene and micron-size electrode materials. We first synthesize a new type of freestanding LIB anode composed of micron-sized Si (mSi) particles wrapped by giant nitrogen-doped graphene (mSi@GNG) film. High ICE (>85%) and long cycle life (more than 80 cycles) are obtained. In the mSi@GNG composite, preferential formation of a stable solid electrolyte interphase (SEI) on the surface of graphene sheets is achieved. The formation and components of SEI are identified for the first time by using UV-resonance Raman spectroscopy and Raman mapping, which will revive the study of formation and evolution of SEI by Raman. New mechanism is proposed that the giant graphene sheets protect the mSi particles from over-lithiation and fracture. Such a simple and scalable method may also be applied to other anode systems to boost their energy and power densities for LIB.

Lithium-ion batteries (LIBs) have significantly expanded its applications in recent years, thanks to technological advances. However further applications, especially in the area of electric vehicles and large-scale renewable energy storage, require increase in its energy density^1^,^2^. Silicon has been intensively pursued as the most promising alternative anode material for LIBs because of its high theoretical capacity (about 3579 mAh g^−1^)^3^. However, there are severe problems associated with Si-based anodes that are eager to be solved. Arising from the dramatic volume expansion/contraction during lithium uptake/release, the Si anode materials suffer traumatic capacity fading and the cycle life is limited to a few cycles only. The micron-sized Si (mSi) particles break up into smaller pieces due to the large volume change and lose of mechanical and electrical contacts with the electrode^4^. The use of nano Si (nSi) or nSi/C composite^5^,^6^, which can accommodate volume change much better, can improve the cycle stability to some extent. However nSi possesses a low tap density, which in turn suppresses their volumetric capacity^7^. Furthermore, it is complicated and costly to prepare nSi, and it is even harder to achieve a massive production with controlled shape^8^,^9^. Most importantly, the low initial coulombic efficiency (ICE) because of the high surface area and high risk of the oxidation (forming SiO_x_) make nSi anode difficult for practical usage^10^. On the other hand, mSi with high tap density is readily available in large quantity at low price. It is favored for large scale applications if the capacity fading can be resolved^11^. One approach was recently adopted by N. Ding *et al.*, in which an mSi-carbon composite was formed by coating soft carbon on mSi and adding carbon black as the conducting additive in the electrode^12^. Nevertheless the use of large amount of carbon (40 wt %) would result in the reduction of the energy density due to low specific capacity of the carbon black. Their ICE is also around 65%, too low for any practical usage.

Graphene is a 2D material with outstanding electrical, mechanical, chemical and thermal properties and with specific capacity (~600 mAh g^−1^)^13^,^14^. Various strategies towards preparing of large-size GO and graphene sheets have been developed^15^,^16^. The giant graphene manifested superior reversible capacity and excellent rate capability for electrode material^14^. In particular, the electrochemical performance of graphene can be greatly enhanced by doping or functionalization^17^,^18^,^19^,^20^. Nitrogen-doped graphene (NG) was reported to double its specific charge capacity in comparison to the pristine graphene^21^. N-doping also increases the conductivity on the graphene sheets and hence improves the overall performance of the composite electrodes. Nano Si/Graphene composites have been used to improve the stability of the silicon anode^22^,^23^,^24^,^25^,^26^,^27^. However, the preparation of mSi/graphene composites, in particular the mSi and giant nitrogen-doped graphene (GNG) composites has never been reported. The effect of graphene in protecting nano-Si was popularly pointed to be the enhancement in conductivity and accommodation of volume expansion in previous reports^28^. It was never been indeed researched in detail of the real role of graphene in protecting Si from the electrochemical and physical perspective.

In this study, a free standing mSi@GNG composite film consisting of GNG-wrapped mSi was prepared and characterized. This mSi@GNG composite delivers high specific capacity (548 mAh g^−1^) at high current density (500 mA g^−1^), good cycling stability as well as ultra-high ICE (~85%). What’s more, both the simple structure and no binder or conductive additive features are favored to study the real mechanism of graphene in protecting Si from fading and ICE improvement from the electrochemical perspective. We found from results of electrochemical test that a stable SEI is preferentially formed on graphene surfaces in mSi@GNG composite. UV resonance Raman spectroscopy and Raman mapping also identified SEI formation. HRTEM result further proved the conclusion from Raman and electrochemical analysis. The role of GNG in preventing SEI formation on mSi particles and protecting Si from over-lithiation to form the unstable Li-Si alloys is proposed.

## Results

**Figure 1:**
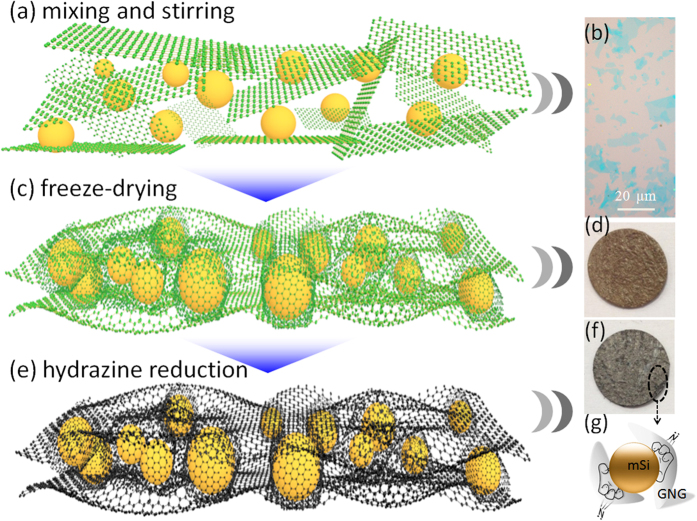
Schematic fabrication process for the mSi@NG composite: (**a**) mSi and GGO in an aqueous solution. (**b**) Optical micrograph of the giant graphene platelets. (**c,d**) freestanding mSi@GGO composite after freeze-drying. (**e–g**) Freestanding mSi@GNG composite film after hydrazine reduction.

The synthesis process of the freestanding mSi@GNG composite is schematically illustrated in Fig. 1. Firstly, giant graphene oxide (GGO) was prepared according to modified Hummers method^14^. The as-prepared GGO aqueous solution was centrifuged to remove un-reacted graphite particles, leaving only graphene thin layers in the solution. The optimized centrifugation conditions to obtain large-sized few-layer graphene have been obtained. Figure 1b displays a typical optical micrograph of the graphene sheets with lateral sizes ranging from a few to several tens of micrometers. Figure S1 shows an atomic force microscopy image of a selected graphene sheet with a thickness less than 5 nm. The video that can be found in the Supplementary Information shows that a large number of thin-layered, large-area GGO sheets were uniformly dispersed on Si substrate. Commercially available mSi (1 ~ 10 micron) was dispersed into the centrifuged GGO solution (Fig. 1a). The mixed solution was then transferred into a culture dish and frozen directly after sonication and stirring for one hour. A spongy dark yellow block of mSi/GGO composite was obtained by freeze-drying under vacuum (Fig. 1c,d). This mSi/GGO composite was pressed to a film using a compression machine. Some SEM photographs of freeze-dried mSi-GGO, compressed mSi-GGO and reduced mSi@GNG was shown in Fig. S2. The micro size porous structure of freeze-dried mSi-GGO was transformed into a dense film under compression force (see tap densities in Supplementary Table S1). A moderate surface area of the mSi@GNG (~87 m^2^ g^−1^) should benefit for initial coulombic efficiency (see Fig. S3). The yellow film was then converted to a black free-standing film via an *in situ* hydrazine vapor treatment at 120 °C^29^. The original functioned GGO groups could be transformed into RGO under the hydrazine monohydrate vapor, while N was simultaneously introduced into GNG (Fig. 1e,f). The successful fabrication of mSi@GNG composite film (~3 at.% for N) can also be confirmed by x-ray photoelectron spectroscopy (XPS) and x-ray diffraction (XRD) measurement (see Figs S4, 5 and associated discussion). The introduction of N into graphene can enhance the adhesion of graphene layer to mSi particles due to a lone pair of electrons of N (see Fig. 1g)^30^. This free-standing mSi@GNG film was directly used as an electrode for lithium ion battery, without binder and conducting additive.

**Figure 2:**
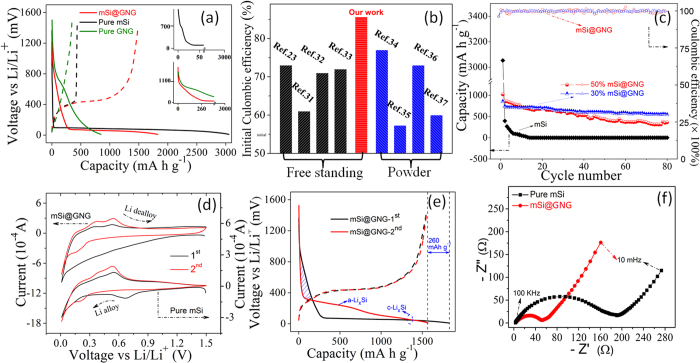
Electrochemical properties: (**a**) Initial charge-discharge curves of pure mSi, pure GNG and mSi@GNG anode with 50% Si at 0.1 A g^−1^ (insets are the enlarged discharge curves of each electrode). (**b**) The initial coulombic efficiency of Si and graphene composite reported recently compared to mSi@GNG composite film electrode. (**c**) Cycling performance of discharge capacity and coulombic efficiency of the mSi@GNG composite film electrodes and pure mSi electrode at 500 mA g^−1^. (**d**) CV curves of the 1^st^ and 2^nd^ cycles of the mSi@GNG composite and pure mSi electrode. (**e**) The 1^st^ and 2^nd^ charge–discharge curves at 0.1 A g^−1^ for mSi@GNG composite. (**f**) Nyquist plots of pure mSi and mSi@GNG electrodes.

The electrochemical performance of the freestanding mSi@GNG composite was tested in a coin-typed cell using Li foil as the reference electrode. Figure 2a shows the first cycle discharge/charge profiles of mSi@GNG, pure mSi and pure GNG electrodes. The first charge capacity is 1551, 437 and 370 mAh g^−1^ for mSi@GNG, pure mSi and pure GNG, respectively. A rather high ICE (85.6%) is calculated for mSi@GNG (the result is fairly reproducible in Fig. S6), which is much higher than those of pure mSi (15%) and pure GNG (35%), as well as those previously reports for Si and graphene composites (see Fig. 2b)^23^,^31^,^32^,^33^,^34^,^35^,^36^,^37^. The insets in Fig. 2a are the enlarged first discharge (lithiation) curves of pure mSi (top), pure GNG (bottom, green) and mSi@GNG (bottom, red), respectively. It can be clearly seen that the SEI formation process starts at 700 mV on the surface of mSi particles and at 1100 mV on both GNG and mSi@GNG, respectively. It is well known that the cathodic reduction at higher potential will be thermodynamically more favorable than that of lower potential. Hence, it can be reasonably assumed that for the first discharge process on mSi@GNG electrode, SEI formation starting from 1100 mV mainly occurs on the GNG surface. Meanwhile, the mSi@GNG anode with 30% Si shows excellent rate performance (Fig. S7), which provides the approximately reversible capacities of 752, 670, and 548 mAh g^−1^ at current density of 100, 200, and 500 mA g^−1^, respectively. As shown in Fig. 2c, the mSi@GNG electrode with 30% Si content shows the best stability, achieving a high specific capacity of 510 mAh g^−1^ after 80 cycles at 0.5 A g^−1^, which is much higher than pure mSi and commercial graphite electrode (calculated against the overall mass of the anode electrode)^38^. Three parallel comparisons, including nano Si and micro Si, small size and giant size graphene, and nitrogen and nitrogen free were performed (see Figs S8–10). Electrochemical performance results show that the mSi@GNG composite possess higher ICE than nano Si@GNG, better cycle stability than small size graphene and micro Si composite, more excellent rate performance than nitrogen free graphene and micro Si composite.

The cyclic voltammetry (CV) curves, obtained at a scan rate of 0.1 mV s^−1^ between 0.05 V and 1.5 V, are shown in Fig. 2d. For pure mSi (see CV curve in Fig. 2d bottom), a principal peak appears at ~0.7 V (vs. Li/Li^+^) in the first cathodic (Li alloying) process and it is attributed to the reaction of the electrolyte and the formation of SEI on the surface of mSi particles^39^. During the 2^nd^ cycle, a small peak at ~0.25 V is observed, which is ascribed to the alloying of crystalline mSi to lithiated amorphous Si, a-Li_x_Si. For the mSi@GNG electrode (see CV in Fig. 2b top), the SEI formation peak of mSi at ~0.7 V is not observed in the first cathordic process while the cathodic current starts to increase at a high potential ~1.1 V and continues to rise up till 0.05 V. In our mSi@GNG electrode structure, the mSi particles are wrapped by GNG sheets and SEI formation mainly occurs on the GNG surface. The sloping cathodic section of the first CV curve for mSi@GNG is similar to those reported in literature for graphene samples^40^,^41^, suggesting the preferential formation of SEI on the surface of GNG layers in the 1^st^ cycle. It is also noted that the initial irreversible loss of 260 mAh g^−1^, as derived from the capacity difference of first discharge (1811 mAh g^−1^) and charge (1551 mAh g^−1^) processes for the mSi@GNG sample (Fig. 2e), is almost equal to the discharge capacity of the mSi@GNG sample in the steep sloping plateau between 1.0 and 0.1 V in the first discharge profile (shaded area in Fig. 2e). It is speculated that the irreversible loss of Li ions is consumed in the formation of SEI on GNG which in turn protects the mSi particles from irreversible loss. Hence the Li ions will first be taken by GNG layers, forming stable SEI before the lithiation of crystalline mSi. The cathodic current in the 2^nd^ cycle decreases significantly on mSi@GNG, which indicates the SEI formation on mSi@GNG during 1^st^ cycle. A peak between 0.1 and 0.3 V, which is broader than that of pure mSi samples, is observed for mSi@GNG in the 2^nd^ cathodic process. This peak is attributed to both the lithiation of graphene (0.16 V)^42^ and a-Li_x_Si (0.25 V)^39^. In the anodic branch, two delithiation peaks at 0.38 and 0.54 V are observed to grow with increasing cycles for both pure mSi and mSi@GNG samples. They are related to the de-alloying of amorphous Li–Si alloys^39^. The Nyquist plots in Fig. 2f show that mSi@GNG electrode possesses a much smaller compressed semicircle than that of pure mSi electrode in the medium-frequency region, which is usually assigned to the charge-transfer resistance, indicating an improvement in the conductivity of the mSi@GNG system due to the presence of GNG^21^.

To further demonstrate the mechanism upon cycles, Raman spectroscopy was conducted experimentally to detect the formation of SEI after lithiation/delithiation. Raman spectroscopy is a useful tool to study carbonaceous and silicon materials, as the line shape and peak position of Raman spectra provide important and accurate information concerning the nature and crystalline quality of materials^43^,^44^. In particular, ultraviolet resonance Raman spectroscopy (UV-Raman) using 325 nm laser light as the excitation source is highly sensitive for the detection of typical SEI compounds such as inorganic carbonates and organic polycarbonates. Visible Raman spectroscopy using 532 nm light source is unable to detect these species whereas the intensity of UV-Raman scattering increases significantly because carbonates (both inorganic and organic) and some aromatic compounds have demonstrated strong absorption in the UV excitation wavelength^45^. A resonance between the excitation wavelength and the part of the molecular structure in the samples results in highly intense Raman scattering^46^,^47^.

**Figure 3:**
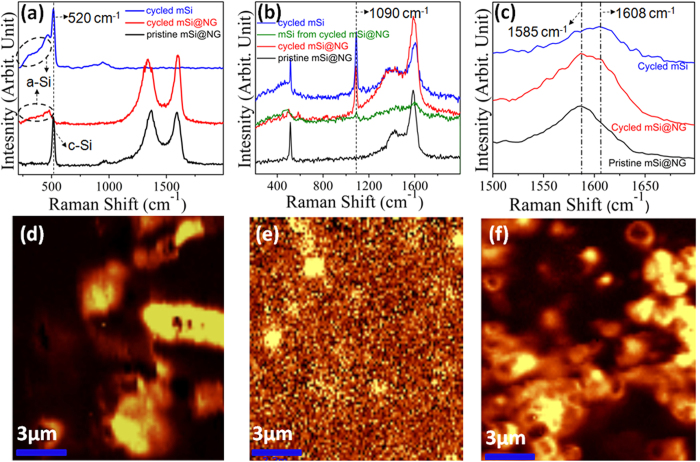
Raman spectra of electrode before and after cycling: (**a**) Raman spectra using 532 nm laser for pristine mSi@GNG film, and cycled mSi and mSi@GNG samples. (**b**) UV resonant Raman spectra using ultraviolet laser (325 nm) for the pristine mSi@GNG film, cycled mSi and mSi@GNG samples. (**c**) Enlarged Raman spectra of Fig. 3b. (**d**) Raman mapping of mSi@GNG. (**e**) Raman mapping of cycled mSi@GNG, integrated from 450–550 cm^−1^, showing as the yellow points. (**f**) Raman mapping of cycled mSi, integrated from 450–550 cm^−1^.

Figure 3a shows the Raman spectra recorded using 532 nm laser excitation. The peak at ~520 cm^−1^ belongs to the characteristic peak of crystalline silicon and it is observed for both the pristine mSi (spectrum not shown) and mSi@GNG samples before lithiation. After 80 cycles of charge/discharge, the crystalline Si peak decays and there appears a broad shoulder peak at ~458 cm^−1^, which is attributed to amorphous silicon. But there are no Raman peaks in the region between 1000 and 2000 cm^−1^ for the cycled mSi sample. The 520 cm^−1^ peak for crystalline Si is much weaker for the cycled mSi@GNG sample while the D (1350 cm^−1^) and G (1580 cm^−1^) peaks of graphene are remarkably strong. No Raman peaks due to SEI compounds can be discerned in Fig. 3a for cycled mSi@GNG samples using 532 nm laser excitation. On the contrary, new Raman peaks at 1090, 1390 and 1605 cm^−1^ are observed in Fig. 3b for both cycled mSi and mSi@GNG samples using the 325 nm UV laser. The peak at 1090 cm^−1^ has been reported to be characteristic of CO_3_^2−^ while the peaks at 1400 and 1605 cm^−1^ are ascribed to polycarbonates^48^,^49^. Both inorganic and organic carbonates are known to be important SEI components^50^,^51^. The polycarbonate peak at 1605 cm^−1^ overlaps with the G band of graphene at 1580 cm^−1^ in the mSi@GNG film (see Fig. 3c). Hence the G peak of GNG in mSi@GNG film appears stronger and shifts slightly to the higher wavenumber after cycling, resulting in the decrease in the apparent I_D_/I_G_ intensity ratio (see also Fig. 3a). This is the first time that SEI formations have been revealed by using UV-resonance Raman spectroscopy. Raman images are formed using the c-Si peak at 520 cm^−1^ to illustrate the degree of Li ion penetration into the m-Si samples. By comparing the Si particles (in the mSi@GNG) Raman image before lithiation shown in Fig. 3d, it is very clear that the c-Si in the mSi@GNG samples break up into little c-Si remains in the Raman image after lithiation (Fig. 3e) indicating that most of the Si particles have been converted to amorphous Si during the charge/discharge process. For the mSi samples, on the other hand, a significant portion remains as crystalline, which means that they partially participate in the lithiation process (Fig. 3f). Our Raman results show that the Si in the m-Si@GNG samples are mostly amorphous after cycling indicating overwhelming majority Si particles are involved in the lithiation process resulting in the high capacity, while the Si particles remain crystalline in bare m-Si samples giving rise to its very poor electrochemical performance.

**Figure 4:**
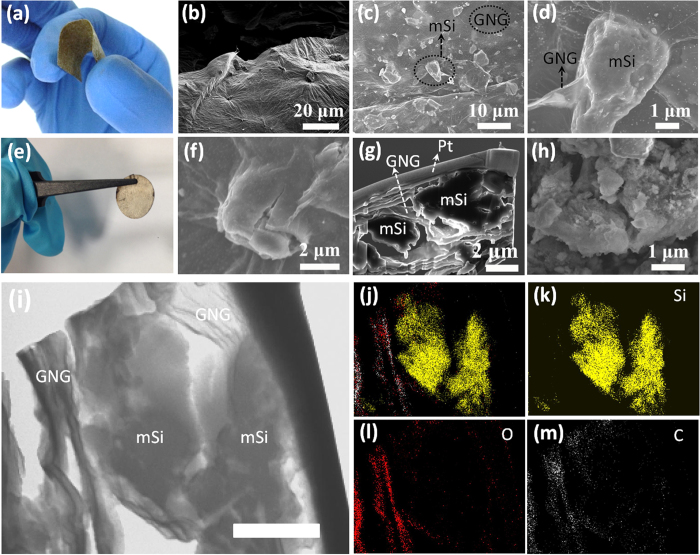
SEM and TEM images of electrodes: (**a**) Photographs of free standing mSi@GNG films. (**b**) Focused Ion Beam (FIB) image of mSi@GNG sample at 52°. (**c,d**) SEM images of the mSi@GNG film. (**e**) Photographs of mSi@NG films after 80 cycles. (**f,h**) SEM images of mSi@GNG and mSi electrode after 80 cycles, respectively. (**g**) FIB-prepared cross section image of mSi@GNG. The Pt coating was deposited to protect the mSi@GNG from ion bombard damage. (**i**) TEM image made by FIB cutting of mSi@GNG electrode after cycles, scale bar: 1 μm. (**j**) Corresponding elements mapping of Si (yellow), O (red) and C (white). (**k–m**) element mapping of Si, O, C, respectively.

The mSi@GNG composite films can bent to near 180 degree (see Fig. 4a) or subjected to long charge-discharge cycles (Fig. 4e). This is benefited from the excellent mechanical flexibility of graphene sheets. As shown in the focused ion beam (FIB) and SEM images in Fig. 4b–d, micro-sized Si particles are uniformly and tightly wrapped/blanketed by graphene sheets. This type of wrapping and blanketing structure can easily accommodate the large volume change of Si during charging/discharging, and achieving similar function as the yolk-shell structure consisting of Si nanoparticles sealed inside a thin and self-supporting carbon shell^52^,^53^. In our structure, the blanketing by GNG sheets makes it much easier to make constant mechanical and electrical contact between the Si particles and GNG sheets even if the Si particles break up into smaller pieces and hence allowing the broken Si particles to continue to participate in the lithiation process. FIB technique was used to prepare a cross section image of the delithiated sample (Fig. 4g), from which micro-sized Si particles are directly seen to be covered by graphene sheets. The smaller Si particle on the left is clearly attached to graphene sheets on the top with void space at the bottom as expected since this is in a delithiated state. The bigger Si particle is attached to the GNG sheets on both top and bottom with void space on both left and right sides. The robust elastic graphene sheets connect Si particles into the network structure, providing excellent electronic connectivity and structure stability for the electrode. This type of flexible structure can also accommodate the large volume change of silicon, enabling the Si particles to expand freely (during lithiation) in the internal void space without breaking the outer graphene shell even after 80 cycles (see Fig. 4f). The GNG sheets also protect the m-Si from direct contact with electrolyte so that the SEI is formed on the GNG sheets and not on the m-Si surfaces, hence resulting in a stabilized SEI by protecting electrolyte re-exposing to fresh surfaces of mSi even if it breaks up and which otherwise causes the continuous growth of SEI. On the contrary without GNG protection, pure mSi particles are seen to break down to smaller pieces (Fig. 4h). These results were obtained in SEM and TEM images before/after lithiation (Fig. S11). To further demonstrate that SEI is mainly formed on NG sheets, rather than on mSi particles in mSi@GNG samples, HRTEM and EDX elemental mapping were performed on an mSi@GNG sample after 80 discharge/charge cycles (see Fig. 4i–m). In Fig. 4I,j, micro Si particles are attached to grapheme sheets with void space between them. Because almost no carbon and oxygen signals appeared between graphene and silicon, there is no SEI formed on the silicon surface (see Fig. 4j). On the contrary, the carbon and oxygen signals obviously coincide in the area of graphene sheets (see Fig. 4k–m), indicating that SEI is preferentially formed on graphene sheets.

**Figure 5:**
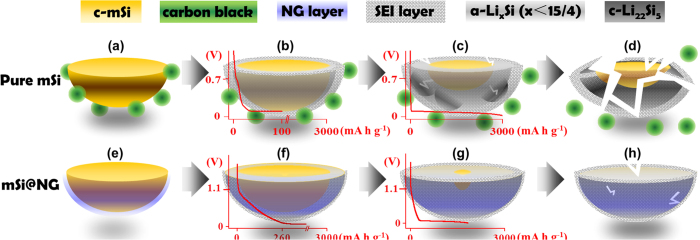
Mechanism of lithium ion storage on pure mSi and mSi@GNG: (**a**) pure mSi particle and conducting additives (e.g. carbon black). (**b**) SEI formation on the surface of pure mSi particles, occurring between 0.7 and 0.05 V during the initial discharge process. (**c**) Further lithiation at 0.05 V. Inhomogeneous point contact between mSi and conducting additives could cause non-uniform lithiation, resulting in stress concentration and the coexistence of c-mSi and meta-stable c-Li_15_Si_4_. (**d**) Broken lithiated mSi due to crystallization of Li_15_Si_4_ and stress-caused cracking after cycles. (**e**) mSi particle and GNG. (**f**) SEI formation on the surface of NG, occurring 1.1 V during the initial discharge process. (**g**) Further lithiation at 0.05 V. homogeneous contact between mSi and GNG could cause uniform lithiation. (**h**) Lithiated mSi@GNG due to no crystallization of Li_15_Si_4_ and no cracking after cycles.

Based on the above results of electrochemical analysis and structural characterization, we can conclude that giant N-doped graphene sheets play several critical roles in improving the electrochemical properties of the mSi@GNG composite, especially the high ICE, good cycleability and good mechanical property as compared to GNG-free mSi. Its mechanism can be inferred and schematically described in Fig. 5 below. For a pure mSi electrode consisting of mSi particles and conducting carbon black (Fig. 5a), SEI forms directly on the surface of mSi during the initial discharge process at 0.7 V (see Fig. 5b). Further discharge at low potential (~0.05–0.1 V) leads to the formation amorphous lithium-silicon alloy a-Li_x_Si and even meta-stable c-Li_15_Si_4_, together with c-Si at the center (Fig. 5c). The very large volume expansion (~300%) during lithiation results in a large structural stress at the Li_x_Si/Si interface that breaks up the particle (Fig. 5c). The partial broken pieces that lost electrical contact with the electrode will cease to participate in the lithiation process and will lead to fade capacity of Si anode. This is the main reason for the very low first cycle efficiency shown in Fig. 2f. As cycles continue, more break-up occurs and thicker SEI forms, both contribute to the further decrease in capacitance. For our mSi@GNG composite electrode on the other hand (Fig. 5e), SEI forms on the surface of the GNG sheets starting at high potential of 1.1 V when discharge (see Fig. 5f). Lithiation of Si particles occur at a lower potential and is mediated by the graphene layer as the Li ions has to go through the graphene layers in order to reach Si. It is important to note that graphene has a much lower capacity at low electric potential as compared to Si as shown in Fig. 2a, which means graphene will only be able to take in small amount of Li ions at low potential and it acts to prevent Si from over-charging (Fig. 5h).

## Discussion

In summary, a freestanding mSi@GNG composite film was successfully fabricated by a simple, low cost and practically scalable method. Especially a high initial coulombic efficiency 85% and good cycling stability of macro Si-based electrode can be achieved that may meet the demand of industrial production for high performance LIBs. These improvements can be ascribed to the synergy effect between GNG and mSi, the mechanism is proposed by electrochemical analysis, and the formation and components of SEI are identified for the first time by Raman analysis. The GNG layers wrapping on mSi can form a wrap/blanket structure which serves as an excellent matrix to accommodate the volume expansion upon lithiation/extraction as well as provides a conducting network to maintain the conductivity of the whole electrode. Micro Si is the major component responsible for large reversible capacitance. GNG sheets uptake Li ions preferentially during the initial lithiation process, forming stable SEI on the GNG surface which can protect mSi from forming dense SEI and avoid/mitigate over-lithiation of mSi in the substantial cycles. Without forming crystalline, the lithiated Si can remain stable for the following cycles. We believe that our mSi@GNG composite with low production cost and excellent performance could be a potential candidate to develop high-performance electrochemical energy storage devices. The protection mechanism of graphene sheets in prolonging the cycle life of Si anode may find wide applications in the development of alternative anode materials.

## Methods

### Preparation of giant graphene oxide (GGO)

Giant graphene oxide (GGO) was prepared according to modified Hummers method^14^. A small amount of expandable graphite was sealed in a glass vial. The vial was then heated in a microwave oven for ~15 seconds under ambient atmosphere using a commercial microwave oven to form worm-like graphite (WG). Giant graphene oxide (GGO) was prepared from WG according to modification of the Hummers. Three grams of WG was added to concentrated sulfuric acid (400 ml) at 0 °C. Then 6 g of KMnO_4_ was added slowly until dissolved. The reaction was kept at 35 °C for 2 hours. Again, the mixture was added to 400 ml de-ionized water and temperature rises to 90 °C for 1 h. It reacts violently with organic material and must be treated with extreme caution. The sediment was decanted and the remaining solution was then centrifugated and washed with a total of 500 ml of 5% HCl solution three times, then washed with DI water 10 times.

### Synthesis of nitrogen-doped graphene wrapped micro-sized Silicon (mSi@GNG) film composite

In a typical synthesis of mSi@GNG composite, 400 mg mSi particles (1 ~ 10 micron) were dispersed in 200 mL previous GGO solution by sonication and strong stirring in a water bath. The as-generated brown dispersion was lyophilized (freeze-drying) directly to get a dark yellow block of mSi/GGO composite, followed by an *in situ* hydrazine vapor reduction at 120 °C for 2 h in a Teflon-lined autoclave contained 500 μL hydrazine monohydrate. After cooling to normal temperature, the composite was washed by DI water for 4 times to remove the adsorbed hydrazine and other by-products. Finally, the product was dried at 100 °C under vacuum for 2 h to get the mSi@GNG composite film.

### Material characterization

Scanning electron microscopy images were obtained by a field-emission scanning electron microscope (FE-SEM JEOL JSM-6700 F; JEOL, Tokyo, Japan). Raman spectra were recorded using a WITEC-CRM200 Raman system with excitation source 532-nm and 325-nm lasers (WITEC, Germany). The Si peak at 520 cm^−1^ was used as a reference to calibrate the wavenumber. X-ray diffraction (XRD) studies were performed on a Bruker D8 ADVANCE XRD (Bruker AXS, Germany). X-ray photoelectron spectroscopy (XPS, PHI 5700) and transmission electron microscopy (TEM, JEM-2010, 200 kV) were also used.

### Electrochemical measurements

The electrochemical tests were carried out using a coin-type cell (CR 2032). The mSi@GNG composite film with a diameter of 12 mm was directly used as the electrode. The mass of the mSi@GNG composite electrode is ~3 mg. The battery cells were assembled in an argon-filled glove box with the metallic lithium foil as the counter electrode, 1 M LiPF_6_ in ethylene carbonate (EC)–dimethyl carbonate (DME) (1:1 in volume) as the electrolyte, and a polypropylene (PP) film (Cellgard 2400) as the separator. For pure mSi and pure GNG, the working electrodes were prepared by mixing 80 wt% active materials, 10 wt% acetylene black and 10 wt% polyvinylidene fluoride binder dissolved in N-methyl-2-pyrrolidinone. After coating the above slurries on Cu foils, the electrodes were dried at 80 °C in vacuum for 2 h to remove the solvent before pressing. Then the electrodes were cut into disks (diameter of 12 mm) and dried at 100 °C for 24 h in vacuum. The battery fabrication method was identical with that of mSi@GNG composite electrode. The CV measurements were carried out using a Solartron 1287 electrochemical workstation at a scanning rate of 0.1 mV s^−1^. For electrochemical impedance spectroscopy (EIS), the amplitude of the sine perturbation signal was 5 mV, and the frequency was scanned from the highest (100 kHz) to the lowest (10 mHz). Galvanostatic charge discharge cycles were tested on LAND CT2001A electrochemical workstation at a current density of 500 mA g^−1^ between 0.05 and 1.5 V vs Li^+^/Li at room temperature.

## Additional Information

**How to cite this article**: Liu, X. *et al.* The roles of lithium-philic giant nitrogen-doped graphene in protecting micron-sized silicon anode from fading. *Sci. Rep.* **5**, 15665; doi: 10.1038/srep15665 (2015).

## Supplementary Material

Supplementary Information
