# Supplementary material for: The roles of lithium-philic giant nitrogen-doped graphene in protecting micron-sized silicon anode from fading
Source: Sci Rep. 2015 Oct 26;5:15665. doi: 10.1038/srep15665 (PMC4620504; doi:10.1038/srep15665)
Supplement: Supplementary Information [file srep15665-s1.doc]

**Supplementary Information**

**The roles of lithium-philic giant nitrogen-doped graphene in protecting micron-sized silicon anode from fading**

*By Xiaoxu Liu^#^, Dongliang Chao^#^,* Q*iang Zhang,* *Hai Liu, Hailong Hu, Jiupeng Zhao, Yao Li, Yizhong Huang*, Jianyi Lin* & Ze Xiang Shen**

^#^These authors contributed equally to this work.

*Correspondence and requests for materials should be addressed to Y.H. (YZHuang@ntu.edu.sg), J.L. (lijy@ntu.edu.sg) or Z.X.S (zexiang@ntu.edu.sg)

**Figure S1.** AFM image of the GGO sheets.


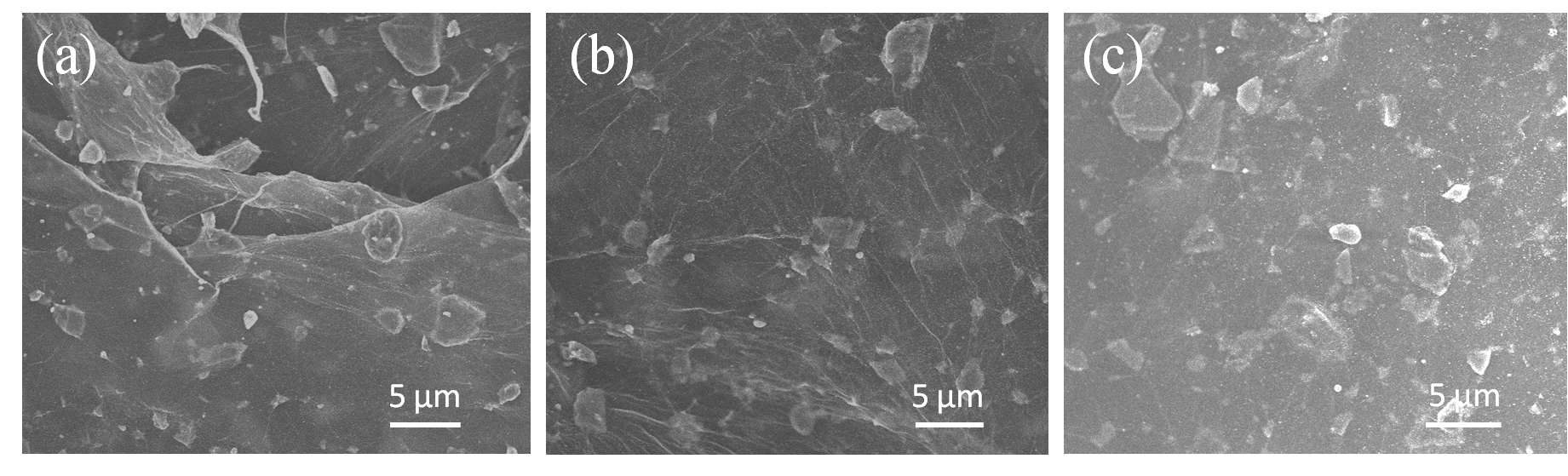


**Figure S2.** (a)SEM images of the mSi@GNGO film. (b) SEM images of compressed mSi@GNGO film. (c) SEM images of reduced mSi@GNGO film.

**Table S1. Tap density and thickness of micro size Si and graphene composite films.**

| **Name of sample** | **Thickness (µm)** | **Tap density (g/cm^3^)** |
| --- | --- | --- |
| ***mSi-GNGO*** | 1028 | 0.145 |
| ***Compressed mSi-GNGO*** | 105 | 1.55 |
| ***reduced mSi@GNG*** | 108 | 1.51 |


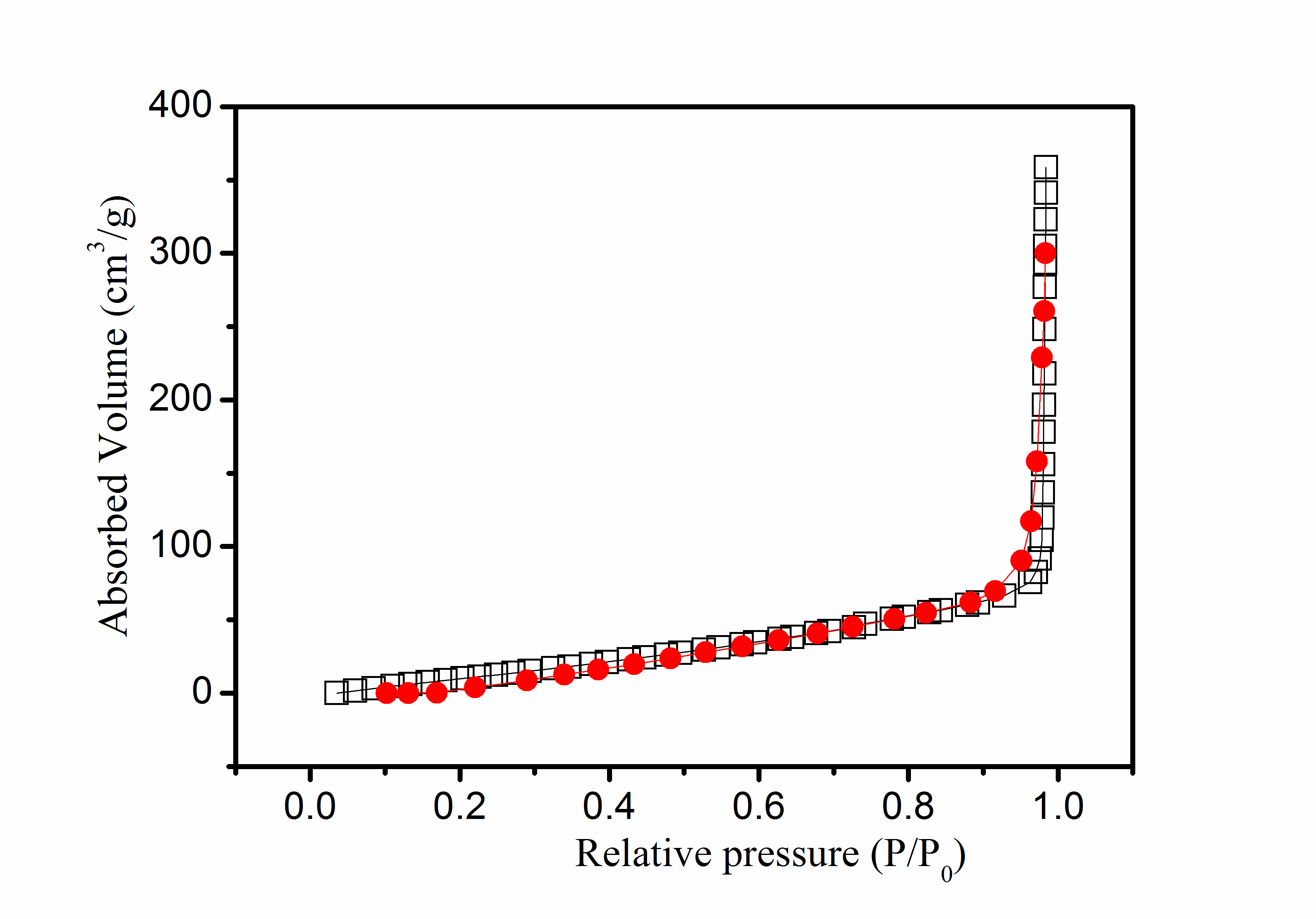


**Figure S3.** N_2_ adsorption/desorption isotherms of mSi@GNG showing a surface area ~87 m^2^/g.

**Discussion on XPS and XRD (Figure S4 and S5)**

To confirm the nitrogen-doping of graphene in the mSi@GNG composite, XPS studies were carried out. As shown in Fig. S3a, the spectrum exhibits peaks at ~101, 284, 400 and 533 eV, corresponding to Si2p, C1s, N1s and O1s, respectively, indicating the incorporation of nitrogen into the graphene. The atomic percentage of nitrogen is calculated about 3 at% of the mSi@GNG composite film. A narrow scan XPS spectrum of the N2p peak further confirms the existence of N. It’s seen that the N1s peak can be resolved into two components centered at 399.5 and 400.5 eV, assigned to pyridinic and pyrrolic nitrogen species, respectively. This is consistent with the theoretical prediction and experimental conclusions by other researchers. The X-ray diffraction patterns of mSi particles and mSi@GNG composite film are shown in Fig. S4. The observation of three sharp Si peaks of (111), (220), and (311) indicates the crystalline structure before and after the fabrication process.


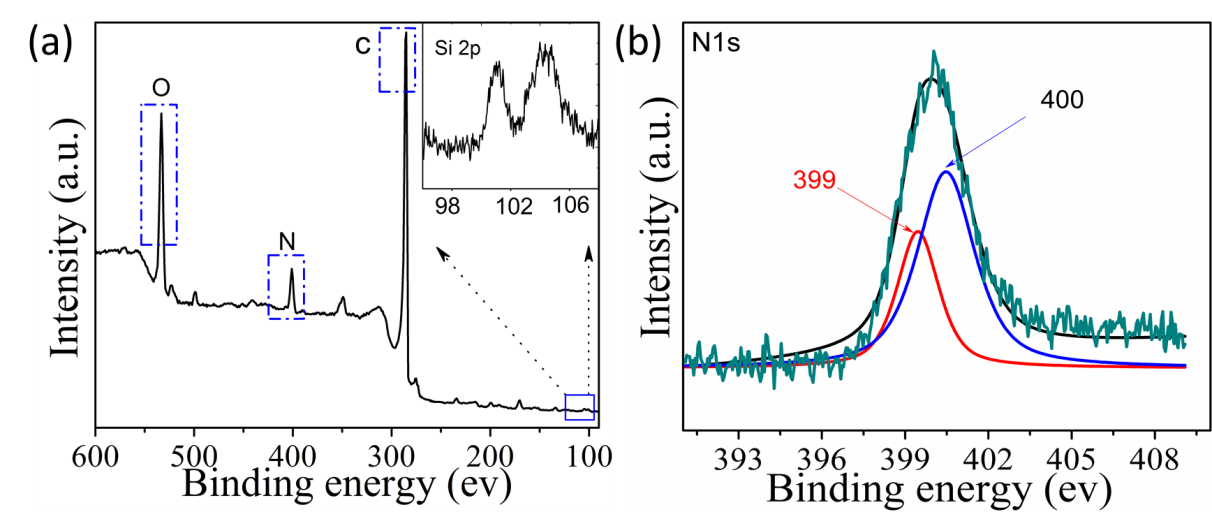


**Figure S****4.** (a) XPS spectrum of mSi@GNG composite film. (b) Narrow-scanned N1s spectrum of the composite film.


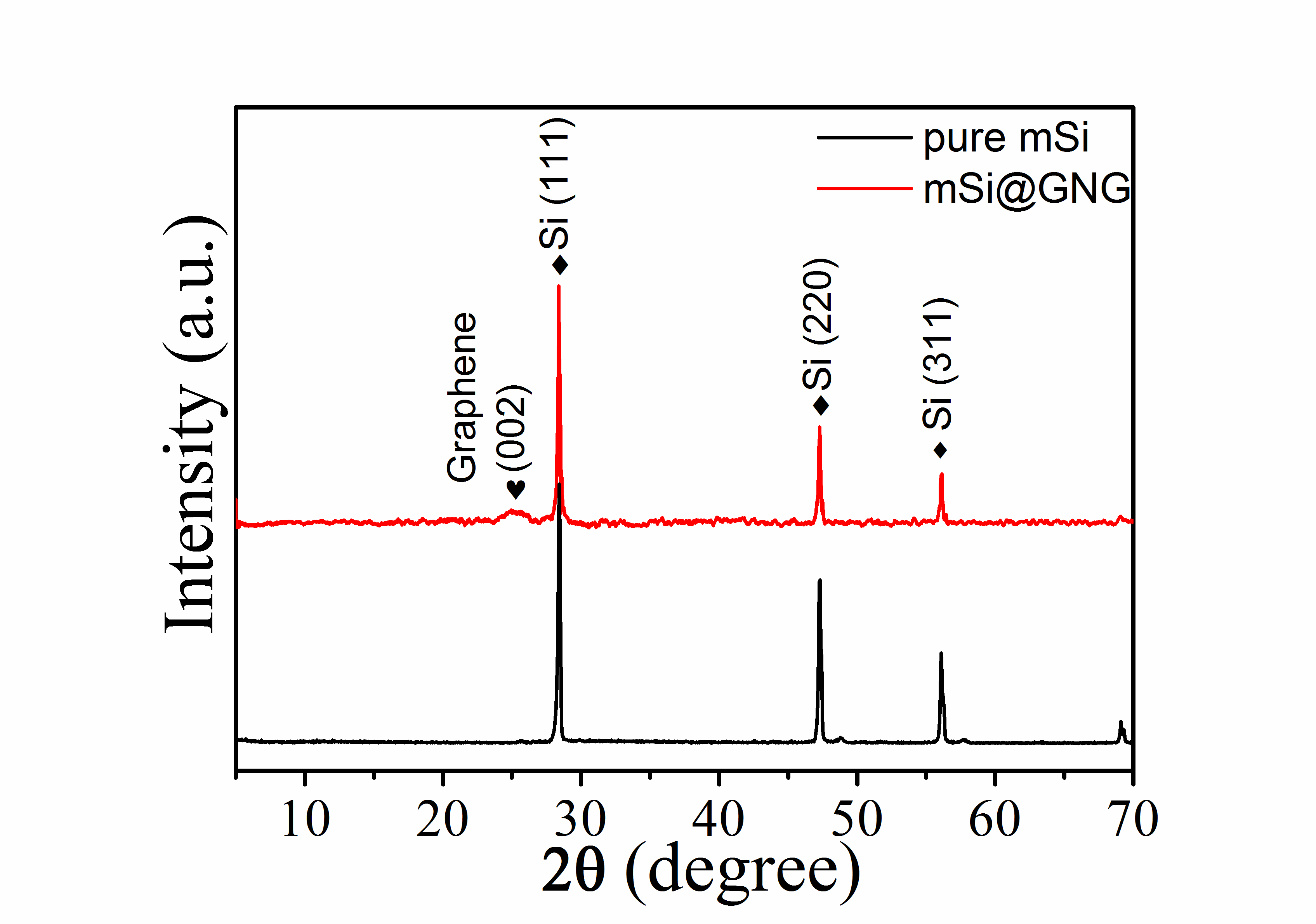


**Figure S5.** XRD patterns of the mSi@GNG composite film and pure micro-silicon powder.


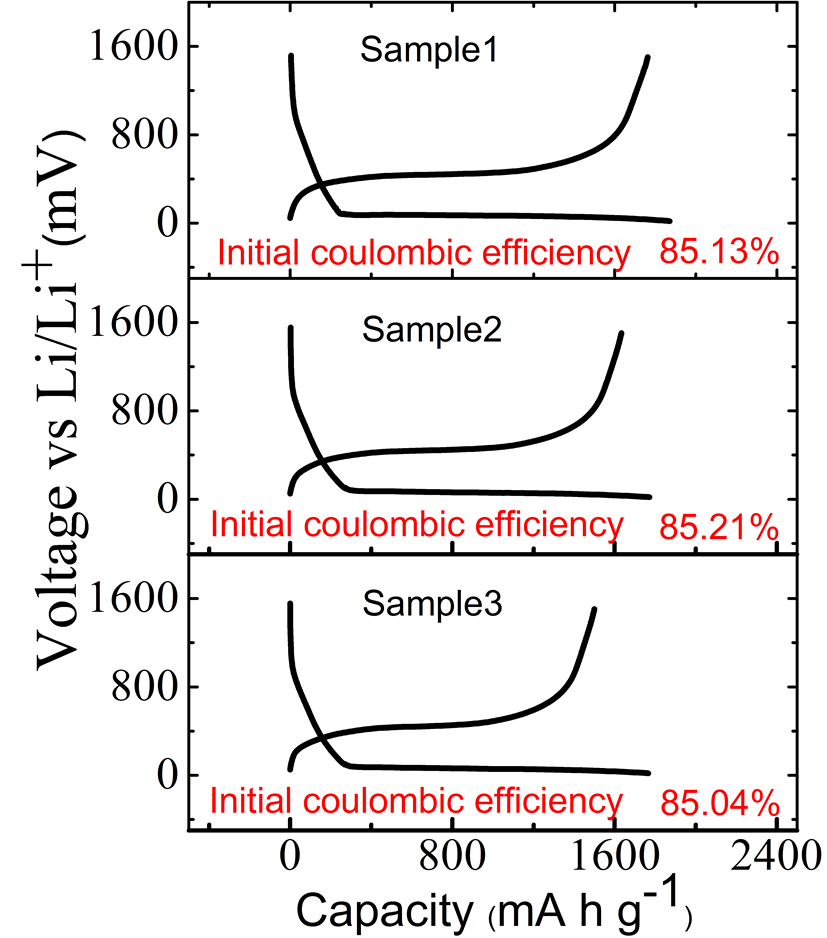


**Figure S6. ICE reproducibility.** Initial coulombic efficiency of three mSi@GNG composite samples.

**Figure S7.** The rate capability of mSi@GNG composite with 30% mSi.


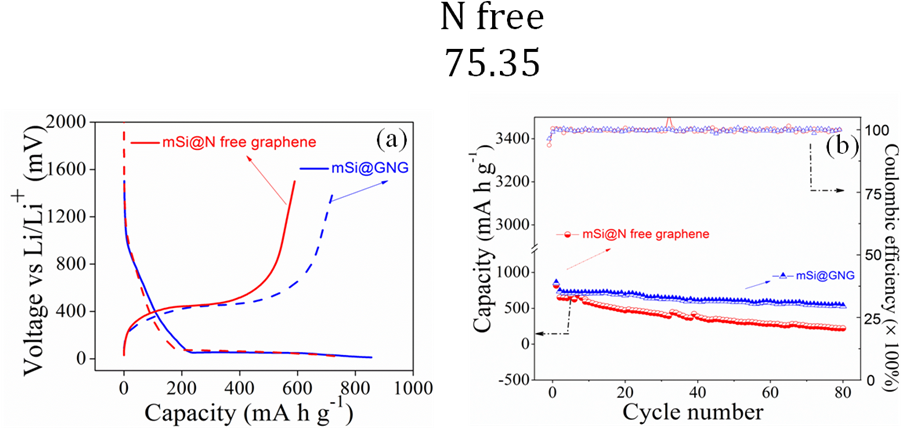


**Figure S8.** (a) The initial discharge/charge curves of mSi@GNG and mSi@N free graphene composite samples with 30% mSi. (b) Cycling performance of discharge capacity and coulombic efficiency of the mSi@ GNG and mSi@N free graphene composite at 500 mA g^-1^.


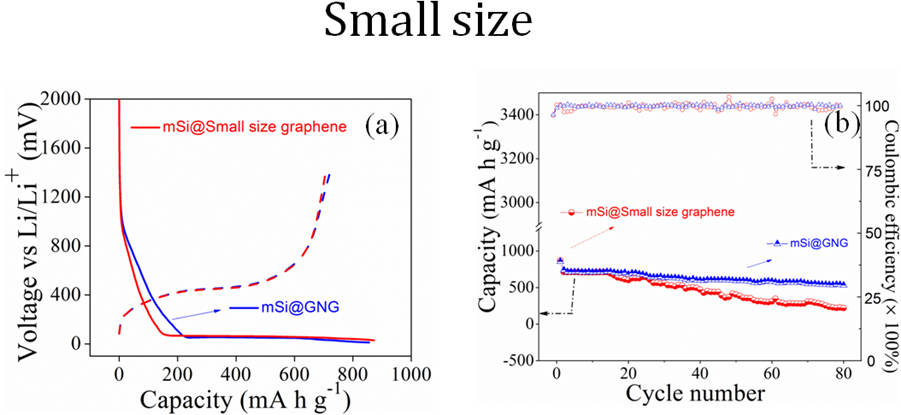


**Figure S9.** (a) The initial discharge/charge curves of mSi@GNG and mSi@ small size graphene composite samples with 30% mSi. (b) Cycling performance of discharge capacity and coulombic efficiency of the mSi@GNG and mSi@ small size graphene composite with 30% mSi at 500 mA g^-1^.


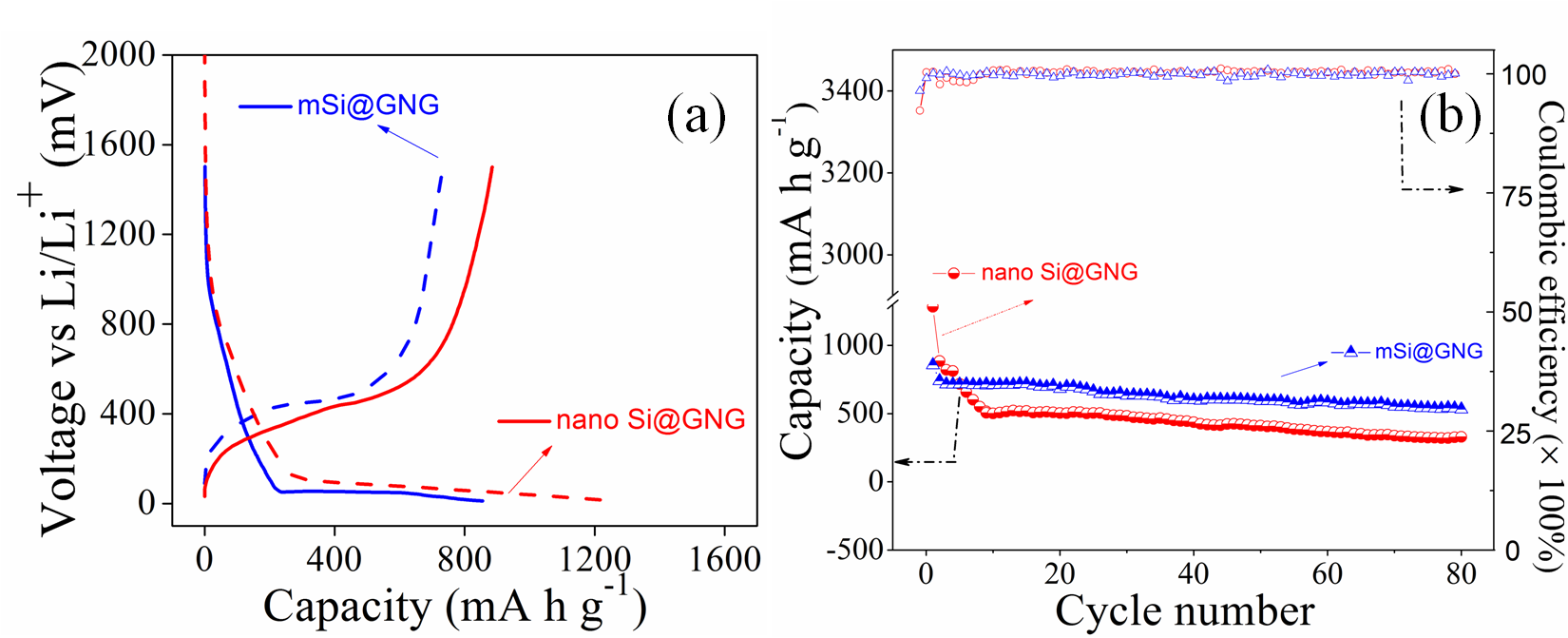


**Figure S10.** (a) The initial discharge/charge curves of mSi@GNG and nano-Si@GNG composite samples with 30% Si. (b) Cycling performance of discharge capacity and coulombic efficiency of the mSi@GNG and nano-Si@GNG composite with 30% Si at 500 mA g^-1^.

**Figure S11.** (a) SEM image of the mSi@GNG anode after cycles; (b) TEM image of the mSi@GNG anode after cycles.

The behaviors of mSi@GNG and pure GNG (in Fig. S12 a) during the first and second charge/discharge processes look very alike. They are obviously different from those of pure mSi (Fig. S12 b), which appears to support the above assumption that the irreversible initial loss is related to the SEI formation on GNG. The capacity for the 2^nd^ discharge of mSi@GNG remains high (1510 mAh g^-1^) due to the protection of SEI on GNG. Without the SEI on NG, pure mSi in the 2^nd^ discharge reduces its capacity substantially to a low value of 480 mAh g^-1^ only. the first and second charge profiles of mSi@GNG almost coincide, with a flat plateau of de-lithiation around 0.4 V, a potential at which the delithiated amorphous silicon is formed. The amorphous de-lithiated silicon as well as the remaining (non-lithiated in the first discharge process) crystalline mSi is lithiated in the following discharging processes, resulting in gradual sloping plateaus between 0.3 and 0.05 V (see Fig. S6b). The results are in good agreement with reported literature.

**Figure S12.** The 1^st^ and 2^nd^ charge–discharge curves at 0.1 A g^-1^ for (a) pure GNG and (b) pure mSi.

**Reference**

[1] X. Liu, D. Zhan, D. Chao, B. Cao, J. Yin, J. Zhao, Y. Li, J. Lin, Z. Shen, Journal of Materials Chemistry A 2014, 2, 12166.

[2] X. S. Zhou, L. J. Wan, Y. G. Guo, Advanced materials 2013, 25, 2152.

[3] G. Q. Wang, W. Xing, S. P. Zhuo, Electrochim Acta 2013, 92, 269; X. R. Wang, X. L. Li, L. Zhang, Y. Yoon, P. K. Weber, H. L. Wang, J. Guo, H. J. Dai, Science 2009, 324, 768.

[4] M. N. Obrovac, L. J. Krause, Journal of the Electrochemical Society 2007, 154, A103.
